# Liraglutide attenuates high glucose-induced abnormal cell migration, proliferation, and apoptosis of vascular smooth muscle cells by activating the GLP-1 receptor, and inhibiting ERK1/2 and PI3K/Akt signaling pathways

**DOI:** 10.1186/s12933-015-0177-4

**Published:** 2015-02-07

**Authors:** Lili Shi, Ye Ji, Xiaoyan Jiang, Lihong Zhou, Ying Xu, Yanbo Li, Wei Jiang, Ping Meng, Xiaomin Liu

**Affiliations:** Department of Endocrinology, The First Affiliated Hospital of Harbin Medical University, Harbin, Heilongjiang China; Department of Orthopedics, The Second Affiliated Hospital of Harbin Medical University, Harbin, Heilongjiang China

**Keywords:** Akt, ERK1/2, Glucagon-like peptide receptor, High glucose, Liraglutide, Vascular smooth muscle cells

## Abstract

**Background:**

As a new anti-diabetic medicine, Liraglutide (LIRA), one of GLP-1 analogues, has been found to have an anti-atherosclerotic effect. Since vascular smooth muscle cells (VSMCs) play pivotal roles in the occurrence of diabetic atherosclerosis, it is important to investigate the role of LIRA in reducing the harmful effects of high-glucose (HG) treatment in cultured VSMCs, and identifying associated molecular mechanisms.

**Methods:**

Primary rat VSMCs were exposed to low or high glucose-containing medium with or without LIRA. They were challenged with HG in the presence of phosphatidylinositol 3-kinase (PI3K), extracellular signal-regulated kinase (ERK)1/2, or glucagon-like peptide receptor (GLP-1R) inhibitors. Cell proliferation and viability was evaluated using a Cell Counting Kit-8. Cell migration was determined by Transwell migration and scratch wound assays. Flow cytometry and Western blotting were used to determine apoptosis and protein expression, respectively.

**Results:**

Under the HG treatment, VSMCs exhibited increased migration, proliferation, and phosphorylation of protein kinase B (Akt) and ERK1/2, along with reduced apoptosis (all *p* < 0.01 *vs.* control). These effects were significantly attenuated with LIRA co-treatment (all *p* < 0.05 *vs.* HG alone). Inhibition of PI3K kinase and ERK1/2 similarly attenuated the HG-induced effects (all *p* < 0.01 *vs.* HG alone). GLP-1R inhibitors effectively reversed the beneficial effects of LIRA on HG treatment (all *p* < 0.05).

**Conclusions:**

HG treatment may induce abnormal phenotypes in VSMCs via PI3K and ERK1/2 signaling pathways activated by GLP-1R, and LIRA may protect cells from HG damage by acting on these same pathways.

## Background

Cardiovascular disease remains the leading cause of mortality and morbidity in diabetes mellitus (DM) patients [1,2]. Atherosclerosis is a complication that can be triggered by damage to vascular smooth muscle cells (VSMCs) in DM patients [3]. High glucose (HG) levels in the blood of DM patients often results in an enhanced generation of reactive oxygen species products, which can stimulate the proliferation and migration of VSMCs, causing accumulation of VSMCs in the intima of blood vessels [4,5]. Additionally, abnormal apoptosis of VSMCs has been observed in atherosclerosis and other DM-related cardiovascular diseases [6]. In DM patients, HG inhibits apoptosis of VSMCs by upregulating anti-apoptotic proteins, including Bcl-2, Bcl-xL, and Bfl-1/A1 [7,8]. Overproliferation or reduced apoptosis of VSMCs accelerates the deposition of atherosclerotic plaques in the lining of blood vessels, and induces intimal thickening and vascular remodeling [9]. Strategies preventing HG-induced alterations to VSMC cell migration, proliferation, and apoptosis may represent promising therapies for protecting blood vessels against diabetic atherosclerosis.

Although the mechanisms by which HG influences VSMC migration, proliferation, and apoptosis remain unclear, *in vitro* studies indicate involvement of phosphatidylinositol 3-kinase (PI3K)/protein kinase B (Akt) and extracellular signal-regulated kinase (ERK) pathways [10]. The ERK1/2 cascade functions in several physiologic events, including cellular proliferation, differentiation, and survival, and the serine/threonine kinase Akt plays an essential role in cell proliferation, migration, and protection against apoptosis [11,12]. In animal studies, hyperglycemia can activate the ERK1/2 pathway in aortic VSMCs [13,14], and HG activates ERK1/2 in cultured VSMCs, which could be an essential event in mediating increased proliferation and migration, and reduced apoptosis [13,15-19]. Hyperglycemia may also inhibit apoptosis [16,20,21] and increase proliferation of VSMCs via activating PI3K/Akt [22,23].

Glucagon-like peptide-1 (GLP-1), a gut incretin, modulates glucose-dependent insulin secretion and suppresses the release of glucagon [24]. A large body of evidence indicates that GLP-1 plays an important role in the pathogenesis of diabetic atherosclerosis. Long-term treatment with GLP-1 effectively improves severe obesity, hypertension, and lipid profiles, all of which are critical risk factors in the development of atherosclerosis [25-28]. GLP-1 also has multiple therapeutic effects on the cardiovascular system, improving cardiac function and exerting direct protective effects on cardiomyocytes [29-31], endothelial cells [32,33], macrophages [34-36], and VSMCs [37]. Moreover, animal studies have demonstrated that GLP-1 can significantly inhibit atherosclerotic plaque deposition in arteries, the formation of macrophage-derived foam cells and the adhesion of mononuclear cells in the intima, and attenuate the abnormal expression of CD36 [34,38]. It also prevents vascular remodeling and protects endothelial cells against oxidative stress via ameliorating intima inflammatory reactions [24,39,40].

Although the molecular mechanisms responsible for the effects of GLP-1 in the cardiovascular system are still uncertain, anti-apoptotic effects of GLP-1 on cardiomyocytes involve regulation of the PI3K/Akt and ERK1/2 signaling pathways [31,41-43]. Furthermore, GLP-1 affects human endothelial cell proliferation through phosphorylation of Akt [44]. As these PI3K/Akt and ERK1/2 signaling pathways are also involved in the effects of HG on VSMCs [13-15,19,20,22,23], we hypothesized that they are responsible for the effects of GLP-1 on VSMCs treated with HG.

GLP-1 specifically binds to GLP-1 receptor (GLP-1R) to stimulate the adenylyl cyclase pathway resulting in increased insulin synthesis and release [45,46]. GLP-1R is expressed on VSMCs [47], and platelet-derived growth factor-induced VSMC cell proliferation is significantly inhibited by a GLP-1R agonist (Exendin-4) [48]. However, no efforts have been made to examine the direct effects of GLP-1 on the HG-induced cell migration, proliferation, and apoptosis of cultured VSMCs.

In this study, we investigated the role of liraglutide (LIRA), a GLP-1 analog, in the attenuation of HG-induced VSMC migration, proliferation, and reduced apoptosis. Furthermore, the mechanisms underlying these effects were also studied.

## Methods

### Animals

Male Sprague Dawley rats (*n* = 4; 5–8 wks) were provided by the Laboratory Animal Center of Harbin Medical University, China. All procedures were performed in accordance with guidelines set by the Institutional Animal Care and Use Committee of the First Affiliated Hospital of Harbin Medical University, which is in compliance with the ARRIVE guidelines on animal research [49].

### Reagents

LIRA was purchased from Novo Nordisk (Bagsvaerd, Denmark). The ERK1/2 inhibitor (PD98059), PI3K inhibitor (LY294002), and FITC-conjugated anti-α-smooth muscle actin (α-SMA) monoclonal antibody were obtained from Sigma-Aldrich (St Louis, MO, USA). The GLP-1R antagonist, Exendin (9–39) (Exe(9–39)), was purchased from AnaSpec (San Jose, CA, USA). Trypsin, fetal bovine serum (FBS), and Dulbecco’s modified Eagle’s medium (DMEM) were purchased from Gibco (of Thermo Fisher Scientific, Waltham, MA, USA). Rabbit polyclonal antibodies against ERK1/2, Akt and phosphorylated (p)-Akt, and the mouse monoclonal antibody against p-ERK1/2 were purchased from Santa Cruz Biotechnology Inc. (Dallas, TX, USA). Rabbit polyclonal antibodies against β-actin, Bax, Bcl-2, and caspase-3 were purchased from Abcam (Cambridge, UK). Secondary antibodies were purchased from Vector Labs (Burlingame, CA, USA). Western blot bands were detected using the ECL Advance Western blotting detection kit from GE Healthcare (Chalfont St Giles, UK). Cell Counting Kit-8 was purchased from Dojindo Molecular Technologies (Rockville, MD, USA). Transwell plates were purchased from Millipore (Bedford, MA, USA). Annexin V-FITC Kit was purchased from BD Biosciences (San Jose, CA, USA).

### Cell culture

VSMCs were prepared from thoracic aorta of Sprague Dawley rats as previously described, with minor modifications [50]. Each rat was sacrificed and the whole thoracic aorta was isolated and washed several times in phosphate buffered saline (PBS). After the adventitia and intima were carefully removed, the aortic tissue was cut into small pieces (1 mm^2^). Theses explants were plated into a tissue culture flask and cultured in DMEM supplemented with 15% FBS and maintained in a humidified incubator at 37°C and 5% CO_2_. The cells were passaged by trypsinization and reseeded into new flasks approximately 4–8 times before use in subsequent experiments. VSMCs were identified by α-SMA staining.

### Drug/chemical treatment

All cultures were incubated with serum-free media for 24 h prior to treatment. Cultures of VSMCs were divided into the following groups: control (5 mM glucose); HG (25 mM glucose); LIRA (100 nM; previously described dose [51]); HG + LIRA (25 mM glucose and 100 nM LIRA); osmotic (MG; 25 mM mannitol); HG + LY294002 (25 mM glucose and 50 μM LY294002 [52]); HG + LIRA + LY294002 (25 mM glucose, 100 nM LIRA, and 50 μM LY294002); HG + PD98059 (25 mM glucose and 50 μM PD98059 [53]); HG + LIRA + PD98059 (25 mM glucose, 100 nM LIRA, and 50 μM PD98059); and HG + LIRA + Exe(9–39) (25 mM glucose, 100 nM LIRA, and 200 nM Exe(9–39) [54,55]). All VSMCs cultures were pretreated with the indicated drugs 1 h prior to HG treatment with exception of Exe(9–39) treatment, which was added 30 min prior to HG treatment.

### Cell proliferation assay

Cells were plated in 96-well culture plates and incubated until reaching 80% confluency; the culture media was then replaced with serum-free DMEM and incubated for an additional 24 h. Cells were then treated with the media containing the indicated concentration of various purposely designed chemical(s) for an additional 48 h. Cell proliferation was then assessed using a Cell Counting Kit-8, following the manufacturer’s instructions. Briefly, the colorimetric reagent, 2-(2-methoxy-4-nitrophenyl)-3-(4-nitrophenyl)-5-(2,4-disulfophenyl)-2H-tetrazolium monosodium salt, was added to each sample and incubated for 1 h at 37°C. Proliferation was then assessed by measuring absorbance of each sample at the wavelength of 450 nm.

### Transwell migration assay

Cell migration was determined using the Transwell migration assay as previous described, with minor modification [56]. Briefly, VSMCs were treated with the indicated chemical(s) for 12 h at 37°C. The cells were washed three times with PBS after the culture media was removed and then trypsinized with 0.25% (v/v) trypsin and resuspended in serum-free DMEM at 37°C. These cells were then counted and the upper chamber of each Transwell was seeded with 1 × 10^5^ cells per chamber in 0.2 mL serum-free DMEM. As a chemoattractant, 0.8 mL of DMEM supplemented with 20% FBS was added to the lower chamber of each Transwell. Chambers were incubated for 12 h at 37°C with 5% CO_2_. Cells that migrated to the underside of the Transwell filter were fixed with 4% formaldehyde (w/v) for 20 min at room temperature and then immersed into a hematoxylin staining solution for 15 min. After washing with distilled water, membranes were mounted on glass slides and examined by microscopy at 200× magnification.

### In vitro scratch wound assay

The migration capacity of VSMCs was also characterized using a well-established *in vitro* scratch wound model, with minor modifications [57]. VSMCs were grown to confluence and then subjected to scratching using a 200 μL sterile pipette tip. The scratch wound was allowed to heal for 24 h in the presence of the indicated chemical(s). Micrographs were captured for each sample at 0 and 24 h, and the capacity of VSMC migration was evaluated by measuring the width of the scratch wound at both time points using ImageJ [58].

### Assessment of cell apoptosis

Cell apoptosis was measured using the Annexin V-FITC kit, following the manufacturer’s instructions. Briefly, cells treated with the indicated chemical(s) for 48 h and then harvested by trypsinization. Cells were washed twice by centrifugation and re-suspended in PBS. Cells were then collected and re-suspended in 500 μL of the binding buffer. These cells were then stained with 5 μL of Annexin V-FITC and 5 μL of the propidium iodide staining solution for 15 min at room temperature in the dark. The percentage of Annexin V-FITC- and propidium iodide-positive cells was measured by flow cytometry (FACSAria, BD Biosciences, San Jose, USA).

### Western blot analysis

All cells were collected and lysed in 200 μL radioimmunoprecipitation assay buffer with the protease inhibitor phenylmethylsulfonyl fluoride (100 mM) for 1 h on ice. Subsequently, the lysate was centrifuged at 12,000 × *g* for 5 min at 4°C. The supernatant was collected and the total protein concentration was determined using a bicinchoninic acid kit (Thermo Fisher Scientific). Ten μg of total protein from each sample was separated by electrophoresis using 12% SDS-PAGE gels and transferred onto nitrocellulose membranes. The target proteins were measured using the primary antibodies (1:10,000) and their corresponding secondary antibodies (1:50,000), followed by development with an ECL Advance Western blotting detection kit. β-actin was detected as a loading control. Signals were developed on X-ray films following exposure to ECL advance luminescence. The intensity of each band was quantified using Quantity One 4.62 (Bio-Rad Laboratories, Inc., Hercules, CA, USA). Levels of phosphorylated proteins were determined as a ratio of total protein: p-Akt relative to Akt and p-ERK1/2 relative to ERK1/2.

### Statistical analysis

All results were from three independent experiments and are expressed as mean ± standard deviation. Data were analyzed by one-way analysis of variance using SPSS version 17.0 (SPSS Inc., Chicago, IL, USA). A *p* < 0.05 was regarded as statistically significant.

## Results

### Characterization of VSMCs

**Figure 1:**
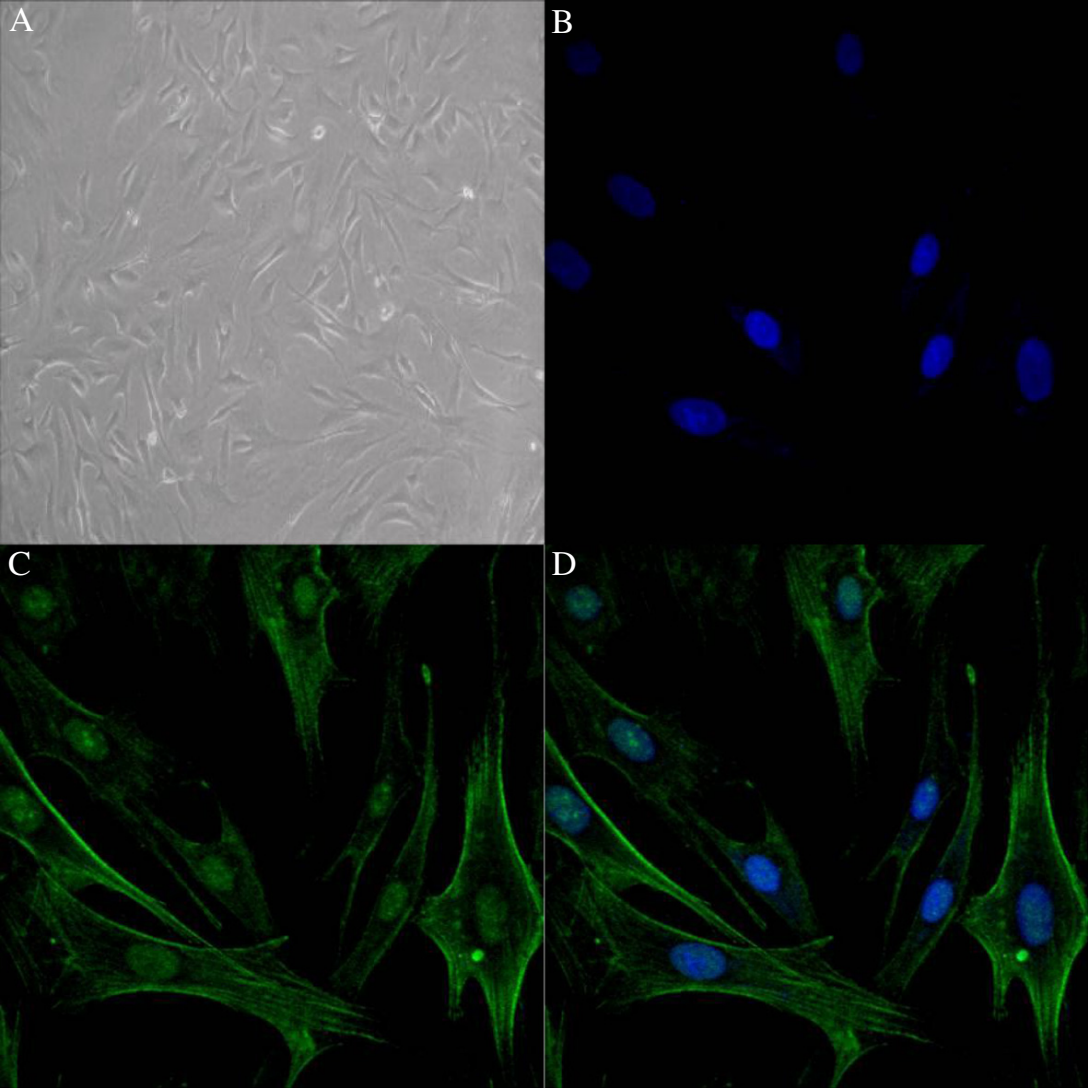
**Identification of vascular smooth muscle cells (VSMCs) in primary cultures. (A)** Primary cultures of VSMCs (phase contrast). **(B)** Nuclear staining with DAPI (fluorescence) **(C)** α-smooth muscle actin staining, a marker of VSMCs (immunofluorescence). **(D)** Co-staining of nuclei and α-smooth muscle actin (fluorescence/immunofluorescence). All images are displayed at 200× magnification.

The isolated VSMCs were identified by α-SMA staining, a VSMC-specific marker. All cultures had comparable numbers of cells and more than 98% of the cells were α-SMA-positive (Figure 1).

### LIRA inhibited HG-induced proliferation of VSMCs

**Figure 2:**
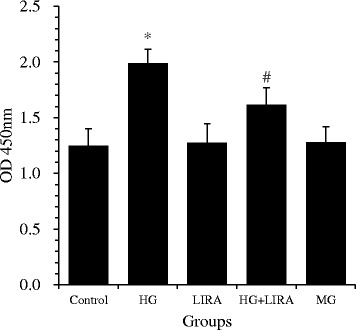
**Liraglutide (LIRA) inhibited high glucose (HG)-induced proliferation in cultured vascular smooth muscle cells.** Cell proliferation was determined using Cell Counting Kit-8. All results are presented as mean ± standard deviation from three independent experiments; ^*^ *p* < 0.01 *vs*. control; ^#^ *p* < 0.01 *vs*. HG. MG = mannitol, osmotic control; OD = optical density.

VSMCs treated for 48 h with HG showed a significant increase in cellular proliferation compared to controls (1.99 ± 0.13 *vs.* 1.25 ± 0.15; *p* < 0.01), and pretreatment with LIRA significantly attenuated this HG-induced VSMC proliferation (1.62 ± 0.15; *p* < 0.01 *vs.* HG) (Figure 2). Treatment of VSMCs with LIRA alone had no effect on cell proliferation, as compared to the control cells. To exclude the possible influence of osmatic change on VSMCs during the HG treatment, mannitol was used in place of glucose, which had no effect on the proliferation of VSMCs.

### LIRA inhibited HG-induced migration of VSMCs

The migration capacity of VSMCs was measured via Transwell migration and *in vitro* scratch assays.

**Figure 3:**
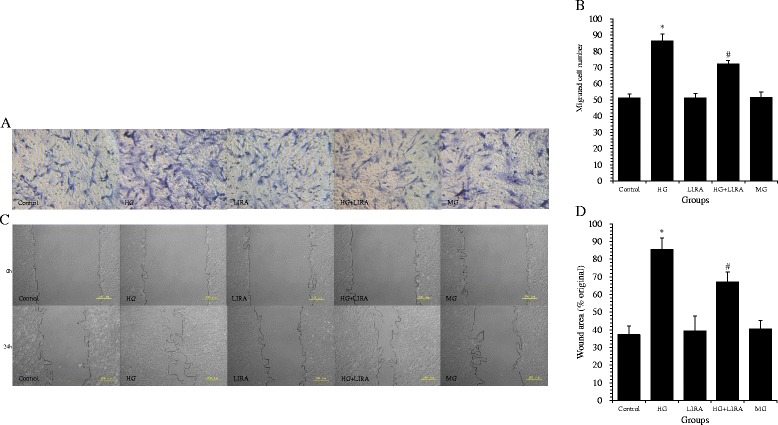
**Liraglutide (LIRA) inhibited high glucose (HG)-induced migration in cultured vascular smooth muscle cells (VSMCs). (A)** Transwell filters were stained with hematoxylin to visualize migrated cells (200× magnification). **(B)** Quantitation of migrated VSMCs in the Transwell migration assay; ^*^ *p* < 0.01 *vs*. control; ^#^ *p* < 0.01 *vs*. HG. **(C)** Confluent VSMCs were wounded and each scratch was imaged at 0 and 24 h after wounding. The wound gaps in each culture were measured to indicate the migration capacity of VSMCs. **(D)** Quantification of the wound area in the scratch assays; ^*^ *p* < 0.01 *vs*. control; ^#^ *p* < 0.05 *vs*. HG. The results are presented as mean ± standard deviation from three independent experiments. MG = mannitol, osmotic control.

A significantly greater number of cells migrated through the Transwell membrane in the HG group than in the control group (86.40 ± 4.22 *vs.* 51.40 ± 2.30 cells; *p* < 0.01). Treatment with LIRA or mannitol alone resulted in no significant increase in migration, as compared to the control group. LIRA pretreatment significantly reduced the number of VSMCs that migrated following HG treatment (72.40 ± 2.07 cells; *p* < 0.01 *vs.* HG) (Figure 3A,B).

The scratch wound assay showed that treatment with HG resulted in significantly more wound healing than the control group (85.61 ± 6.36 *vs*. 37.25 ± 4.78%; *p* < 0.01). LIRA pretreatment was able to attenuate the effects of HG on VSMC migration (67.03 ± 5.61%; *p <* 0.05 *vs.* HG) (Figure 3C,D). Treatment with LIRA or mannitol alone had no effect on scratch width, as compared to the control group.

### LIRA attenuated the inhibitory effect of HG on VSMC apoptosis

**Figure 4:**
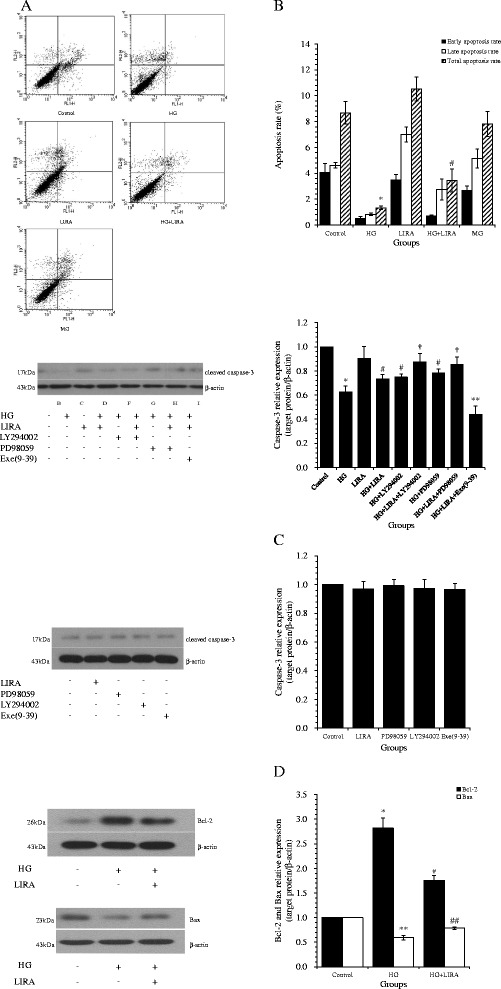
**Liraglutide (LIRA) attenuated the inhibitory effects of high glucose (HG) on apoptosis of cultured vascular smooth muscle cells (VSMCs). (A)** Apoptosis was determined by staining with Annexin V-FITC (x-axis) and propidium iodide (y-axis). For each dot plot, the upper and lower right quadrants represent early apoptotic and late apoptotic cells, respectively. **(B)** Quantification of the apoptosis experiments. Total apoptosis refers to the sum of early and late apoptosis values. Results are expressed as mean ± standard deviation from three independent experiments; ^*^ *p* < 0.01 *vs*. control; ^#^ *p* < 0.05 *vs*. HG. **(C)** Protein expression of cleaved caspase-3 in VSMCs; ^*^ *p* < 0.01 *vs*. control; ^#^ *p* < 0.01 *vs*. HG; ^†^ *p* < 0.05, ^**^ *p* < 0.01 *vs*. HG + LIRA. **(D)** Protein expression of Bcl-2 and Bax in VSMCs; ^*^ *p* < 0.01, ^**^ *p* < 0.01 *vs*. control; ^#^ *p* < 0.01, ^##^ *p* < 0.01 *vs*. HG. Protein expression was normalized to β-actin. The results from three independent experiments are presented as mean ± standard deviation.

Treatment with HG for 48 h significantly reduced the number of apoptotic VSMCs compared to controls (1.34 ± 0.12 *vs*. 8.67 ± 0.87%; *p* < 0.01). Pretreatment of VSMCs with LIRA in HG conditions resulted in a higher rate of apoptosis (3.44 ± 0.89%; *p <* 0.05 *vs.* HG) (Figure 4A,B). Treatment with LIRA or mannitol alone resulted in similar numbers of apoptotic cells to the control sample.

To further investigate the mechanism of the HG-induced reduction in apoptosis, the expression of relevant apoptotic proteins was investigated by Western blotting (Figure 4C,D). The anti-apoptotic protein Bcl-2 was significantly upregulated (*p* < 0.01 *vs.* control) and the pro-apoptotic proteins cleaved caspase-3 and BAX were significantly downregulated after HG treatment (*p* < 0.01 *vs.* control). LIRA pretreatment reduced those effects by HG treatment, resulting in increased cleaved caspase-3 and Bax levels, and decreased Bcl-2 levels (*p* < 0.01 *vs.* HG) (Figure 4C,D). PD98059, LY294002 or Exe(9–39) alone had no effect on cleaved caspase-3 expression (all *p* > 0.05 *vs.* control).

### HG activated PI3K/Akt and ERK1/2 signaling pathways in VSMCs

**Figure 5:**
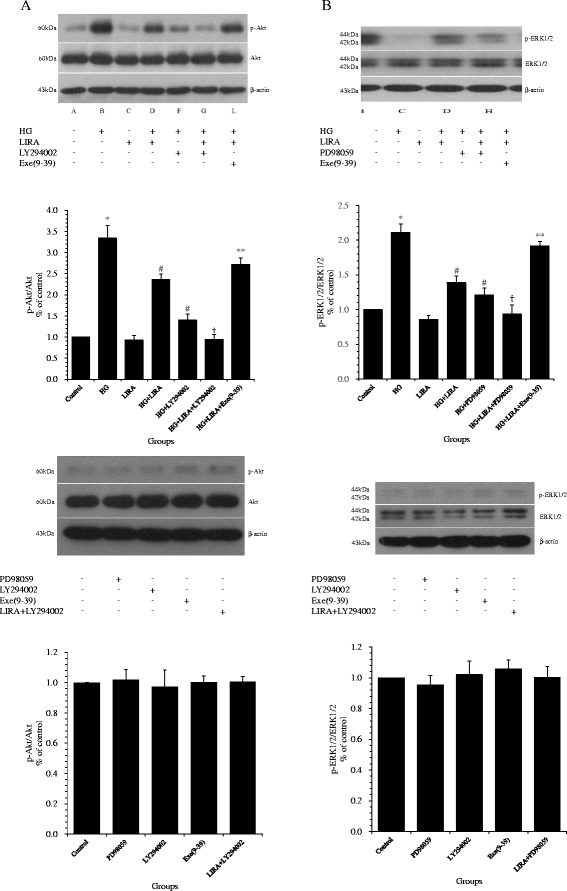
**Liraglutide (LIRA) suppressed the high glucose (HG)-induced activation of PI3K/Akt and ERK1/2 signaling pathways by activating the GLP-1 receptor. (A)** Protein expression of phosphorylated Akt in vascular smooth muscle cells (VSMCs). **(B)** Protein expression of phosphorylated ERK1/2 in VSMCs. Data from three independent experiments are expressed as mean ± standard deviation; ^*^ *p* < 0.01 *vs*. control; ^#^ *p* < 0.01 *vs*. HG; ^**^ *p* < 0.05, ^†^ *p* < 0.01 *vs*. HG + LIRA.

Western blotting analysis showed that HG treatment significantly increased the levels of p-Akt and p-ERK1/2 (both *p* < 0.01 *vs.* control). LY294002, PD98059, and Exe(9–39) had no effect on either p-Akt or p-ERK1/2 expression (all *p* > 0.05 *vs*. control). LIRA plus either LY294002 or PD98059 had no effect on p-Akt or p-ERK1/2 expression (all *p* > 0.05 *vs.* control) (Figure 5A,B).

### LY294002 and PD98059 prevented the HG-induced abnormal VSMC behaviors and the reduction of cell apoptosis

**Figure 6:**
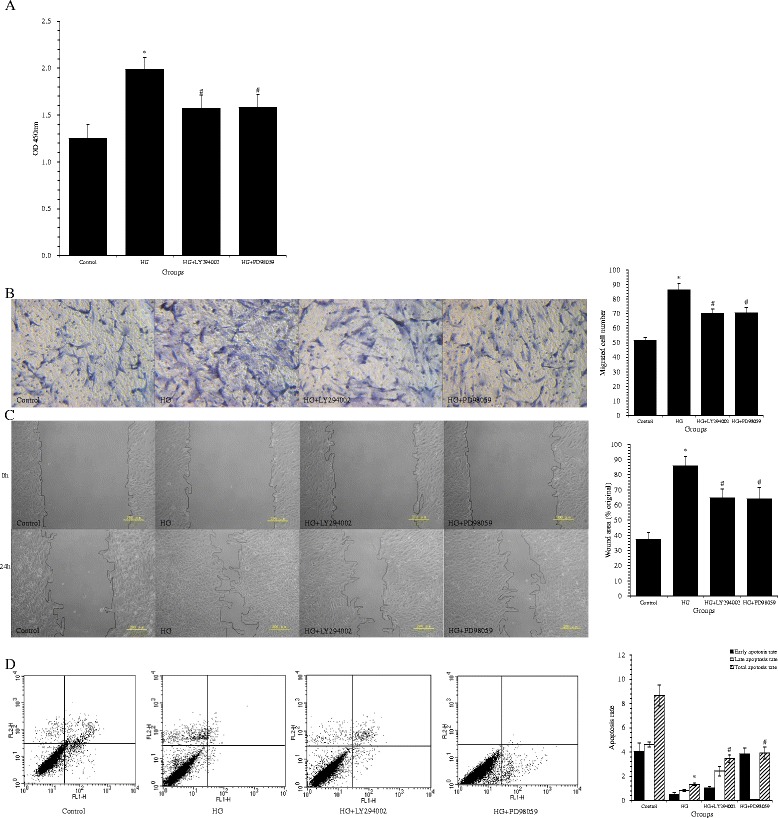
**High glucose (HG) increased vascular smooth muscle cell (VSMC) migration and proliferation, and decreased cell apoptosis in cultures via activation of the PI3K/Akt and ERK1/2 signaling pathways. (A)** VSMC proliferation was determined using Cell Counting Kit-8. **(B)** Representative photomicrographs showing migration of VSMCs in the Transwell migration assay (200× magnification). The relative amounts of migrating cells in all groups are presented. **(C)** Representative photomicrographs showing the migrating cells in the scratch wound assay. **(D)** Apoptosis rates were determined by flow cytometry, with the lower right and upper right quadrants representing early apoptotic and late apoptotic cells, respectively. These values were quantified, and the total apoptotic rate is the sum of the early and late apoptotic rates. Data from three independent experiments are expressed as mean ± standard deviation; ^*^ *p* < 0.01 *vs*. control; ^#^ *p* < 0.01 *vs*. HG. OD = optical density.

Pretreatment with LY294002 or PD98059 reduced VSMC proliferation following HG stimulation (1.57 ± 0.14 and 1.58 ± 0.14, respectively, *vs*. 1.99 ± 0.13; both *p* < 0.01) (Figure 6A), Transwell migration (70.20 ± 3.11 and 70.60 ± 3.16, respectively, *vs*. 86.40 ± 4.22 cells; both *p* < 0.01) (Figure 6B), and wound healing (64.55 ± 6.06 and 63.96 ± 7.71, respectively, *vs*. 85.61 ± 6.36%; both *p* < 0.05) (Figure 6C). LY294002 and PD98059 were also effective in increasing the rate of VSMC apoptosis (3.46 ± 0.31 and 3.93 ± 0.49, respectively, *vs.* 1.34 ± 0.12%; both *p <* 0.01 *vs.* HG) (Figure 6D). Treatment of VSMCs with either LY294002 or PD98059 under HG conditions also resulted in increased protein levels of the cleaved caspase-3 (*p* < 0.01 *vs*. HG) (Figure 4C), and decreased p-Akt and p-ERK1/2 (both *p* < 0.01 *vs.* HG) (Figure 5A,B).

### LIRA exerted beneficial effects on the cultured VSMCs with HG treatment via inhibiting PI3K/Akt and ERK1/2 signaling pathways

**Figure 7:**
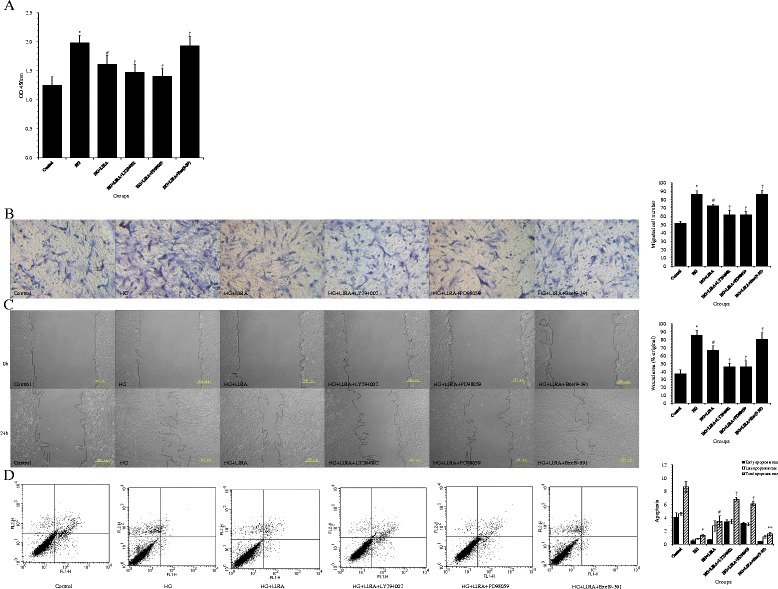
**Liraglutide (LIRA) exerted beneficial effects on cultured vascular smooth muscle cell (VSMCs) by activating the GLP-1 receptor and inhibiting PI3K/Akt and ERK1/2 signaling pathways. (A)** Cell proliferation of VSMCs; ^*^ *p* < 0.01 *vs*. control; ^#^ *p* < 0.01 vs. HG; ^†^ *p* < 0.05 *vs*. HG + LIRA. **(B)** Representative photomicrographs showing migration of VSMCs in the Transwell migration assay (200× magnification). The relative amounts of migrating cells in all groups are presented; ^*^ *p* < 0.01 *vs*. control; ^#^ *p* < 0.01 *vs*. HG; ^†^ *p* < 0.01 *vs*. HG + LIRA. **(C)** Representative photomicrographs show the migrating cells in the scratch wound assay; ^*^ *p* < 0.01 *vs*. control; ^#^ *p* < 0.01 *vs*. HG; ^†^ *p* < 0.05 *vs*. HG + LIRA. **(D)** Apoptosis rates were determined by flow cytometry, with the lower right and upper right quadrants representing early apoptotic and late apoptotic cells, respectively. These values were quantified, and the total apoptotic rate is the sum of the early and late apoptotic rates; ^*^ *p* < 0.01 *vs*. control; ^#^ *p* < 0.05 *vs*. HG; ^†^ *p* < 0.01, ^**^ *p* < 0.05 *vs*. HG + LIRA. Data from three independent experiments are expressed as mean ± standard deviation.

Pretreatment with LIRA effectively reduced the HG-induced protein elevation of p-ERK1/2 and p-Akt (both *p* < 0.01 *vs*. HG) (Figure 5A,B). Furthermore, compared to LIRA alone, co-administration of LIRA with LY294002 or PD98059 on VSMCs under HG synergistically reduced cell proliferation (1.48 ± 0.14 and 1.41 ± 0.13, respectively, *vs*. 1.62 ± 0.15; both *p* < 0.05) (Figure 7A) and migration in the Transwell (61.60 ± 5.41 and 61.80 ± 4.09, respectively, *vs*. 72.40 ± 2.07 cells; both *p* < 0.01) (Figure 7B) and wound healing (46.28 ± 4.24 and 46.02 ± 7.34, respectively, *vs*. 67.03 ± 5.61%; both *p* < 0.05) (Figure 7C) assays, and increased apoptosis (6.81 ± 0.28 and 6.15 ± 0.37, respectively, *vs*. 3.44 ± 0.89%; both *p* < 0.01) (Figure 7D). Furthermore, co-administration of LIRA with LY294002 or PD98059 on HG-treated VSMCs resulted in reduced p-Akt and p-ERK1/2 levels (both *p* < 0.01 *vs*. HG + LIRA) (Figure 5A,B).

### Exe(9–39) abolished the beneficial effects of LIRA on HG-treated VSMCs

Application of the GLP-1R antagonist Exe(9–39) ameliorated the effect of LIRA on VSMCs under HG treatment, including a increased cell proliferation (1.94 ± 0.17 *vs*. 1.62 ± 0.15; *p <* 0.05) (Figure 7A) and migration detected by the Transwell (86.2 ± 4.6 *vs.* 72.4 ± 2.07 cells; *p <* 0.01) (Figure 7B) and wound healing (80.79 ± 7.99 *vs*. 63.07 ± 5.61%; *p* < 0.05) (Figure 7C) assays. Moreover, Exe(9–39) reduced apoptosis (1.54 ± 0.29 *vs*. 3.44 ± 0.89%; *p <* 0.05) (Figure 7D) and cleaved caspase-3 (*p* < 0.01) (Figure 4C) in HG + LIRA-treated cells. Finally, inhibition of GLP-1R increased phosphorylation of ERK1/2 and Akt in HG + LIRA-treated cells (both *p* < 0.05) (Figure 5).

## Discussion

Atherosclerosis is the most common cardiovascular complication of diabetes. Vascular remodeling is an important component in the development of atherosclerosis, and both are closely associated with pathologic changes in VSMCs, including changes in proliferation, migration, and apoptosis [59-61]. Previous studies have suggested that HG concentrations promote these altered cellular behaviors in VSMCs [15,16,22,62-64]. However, these data are inconclusive and a mechanistic understanding is still lacking.

### The involvement of PI3K/Akt and ERK1/2 signaling pathways in the HG-induced alteration of VSMC migration, proliferation, and apoptosis

Previous studies have shown that the ERK1/2 signaling pathway plays an important role in VSMC proliferation [65], and insulin [66,67] or HG [13,14] could facilitate proliferation via activation of this pathway. HG can also induce dynamic changes in VSMCs through activation of the PI3K/Akt pathway [16,20-23]. Therefore, these pathways likely play a crucial role in the tissue damage induced by hypertension and atherosclerosis.

To explore whether these signaling pathways are involved in the HG-induced altered physiology of VSMCs, we first investigated whether HG concentrations altered ERK1/2 and PI3K/Akt activation. We then used specific inhibitors to block these signaling pathways. We show, in agreement with previous studies [13,15,17,23], that HG treatment of VSMCs significantly increases the phosphorylation of ERK1/2 and Akt. Furthermore, inhibition of these enzymes reduces HG-induced cell proliferation and migration. Pretreatment with ERK1/2 or PI3K inhibitors also effectively prevents the ability of HG to reduce VSMC apoptosis.

Thus, our data demonstrate that HG treatment of VSMCs alters cell migration, proliferation, and apoptosis via ERK1/2 and PI3K/Akt signaling pathways. These findings open a new avenue to further explore the underlying mechanism by which HG regulates VSMC cell migration in diabetic patients. Furthermore, our study identifies new research areas that may prove critical in deciphering the pathogenesis of, and provides a novel means to prevent, atherosclerosis in DM.

### LIRA attenuates HG-induced cellular dynamic changes of VSMCs by activating GLP-1R and inhibiting PI3K/Akt and ERK1/2 signaling pathways

GLP-1 analogues and receptor agonists have been used in the treatment of DM, increasing pancreatic protein content and mass via boosting S6 kinase phosphorylation and acinar cell mass [68]. LIRA, a GLP-1 analogue, effectively lowers the body weight of diabetic patients, which was associated with enhancing plasma cardiac natriuretic peptides levels [69]. Long-acting GLP-1 mimetics, such as domain antibodies to serum albumin, can sustain the activation of GLP-1R and reduce myocardial infarct size and injury in acute coronary syndrome [70]. Another GLP-1 agonist, exenatide, increases hydrogen sulfide, carbon monoxide, and nitric oxide production, and then reduces central arterial pressure in an animal model [71]. In carotid endarterectomy of diabetic patients, preoperational use of GLP-1R agonists significantly increases the expression of sirtuin-6, a protein involved in the inflammatory pathway of diabetic atherosclerotic lesions and plaque stabilization [72]. A prospective pilot clinical trial has demonstrated that LIRA decreases carotid intima-media thickness in patients with type 2 diabetes [73].

In this study, we show that HG-induced VSMC alterations are prevented by treatment with LIRA. It is possible that in DM patients, LIRA protects the vascular system against atherosclerotic changes by similar mechanisms. Our results also demonstrate that LIRA by itself does not induce measurable physiologic changes in VSMCs, but only blocks HG-induced effects.

Previous studies have shown that GLP-1 protects cardiomyocytes and endothelial cells against cell apoptosis by activating ERK1/2 and/or PI3K/Akt [31,44,74]. However, our data demonstrate that LIRA inhibits this activation, observed as reduced phosphorylation levels in VSMCs. Moreover, synergistic effects are observed when LIRA pretreatment is combined with ERK1/2 or PI3K inhibitors. Although GLP-1 shows protections on cardiomyocytes and endothelial cells by activating ERK1/2 and/or PI3K/Akt [31,44,74], our data suggest that LIRA exerts the beneficial effects on VSMCs in part via inhibiting ERK1/2 and PI3K/Akt pathways. As VSMCs play a distinct role in the development of diabetic atherosclerosis, the involvement of these signaling pathways may be cell-type specific, which requires further investigation.

Cardiomyocytes, endothelial cells, macrophages, and VSMCs all express the GLP-1R, which mediates the anti-inflammatory and anti-proliferation effects of GLP-1 [31,40,48]. To confirm that the effects of LIRA in HG-treated cells occur via GLP-1R activation, VSMCs were pretreated with Exe(9–39). Indeed, Exe(9–39) treatment abolished the beneficial effects of LIRA treatment, and reversed the suppression of Akt and ERK1/2 activation.

## Conclusions

Collectively, this study shows that HG treatment facilitates migration and proliferation of VSMCs, inhibits cell apoptosis, and increases the phosphorylation of ERK1/2 and Akt. These effects are attenuated by LIRA pretreatment; LIRA treatment reduced HG-induced phosphorylation of ERK1/2 and Akt, suppressed cell migration and proliferation, and increased cell apoptosis. Moreover, inhibitors of ERK1/2 and PI3K prevent the damaging effects of HG on cultured VSMCs, suggesting that HG exerts injurious effects via these signaling pathways. Importantly, we also demonstrate that a GLP-1R antagonist can block the beneficial effects of LIRA on VSMCs exposed to HG. These effects were further enhanced by the co-administration of inhibitors of ERK1/2 or PI3K, demonstrating for the first time that LIRA treatment acts through GLP-1R to regulate these signaling pathways. As LIRA is an effective and safe therapeutic candidate, it may become a promising treatment option in the prevention of diabetic atherosclerosis.

## Abbreviations

HG: High glucose
DM: Diabetes mellitus
GLP: Glucagon-like peptide
GLP-1R: Glucagon-like peptide receptor
LIRA: Liraglutide
VSMC: Vascular smooth muscle cell
ERK: Extracellular signal-regulated kinase
PI3K: Phosphatidylinositol 3-kinase
Akt: Protein kinase B
Exe(9–39): Exendin (9–39)
